# Crystal structure of lutetium aluminate (LUAM), Lu_4_Al_2_O_9_

**DOI:** 10.1107/S2056989020005757

**Published:** 2020-04-30

**Authors:** Rayko Simura, Hisanori Yamane

**Affiliations:** a Institute of Multidisciplinary Research for Advanced Materials, Tohoku, University, 2-1-1 Katahira, Aoba-ku, Sendai, 980- 8577, Japan

**Keywords:** crystal structure, Lu_4_Al_2_O_9_, lutetium aluminate monoclinic (LUAM), rare-earth aluminate, Lu_2_O_3_-Al_2_O_3_ system, single-crystal X-ray diffraction

## Abstract

The crystal structure of the title compound containing lutetium, the last element in the lanthanide series, was determined using a single crystal prepared by heating a pressed pellet of a 2:1 molar ratio mixture of Lu_2_O_3_ and Al_2_O_3_ powders in an Ar atmosphere at 2173 K for 4 h. Lu_4_Al_2_O_9_ is isostructural with Eu_4_Al_2_O_9_ and composed of Al_2_O_7_ di­tetra­hedra and Lu-centered six- and sevenfold oxygen polyhedra. The unit-cell volume, 787.3 (3) Å^3^, is the smallest among the volumes of the rare-earth (*RE*) aluminates, *RE*_4_Al_2_O_9_. The crystal studied was refined as a two-component pseudo-merohedric twin.

## Abstract 2

Single-crystal X-ray structure analysis revealed that Lu_4_Al_2_O_9_ is isostructural with Eu_4_Al_2_O_9_ and contains Lu atoms in six- and sevenfold coordination, together with tetra­hedral Al atoms.

## Chemical context   

In the Al_2_O_3_-Lu_2_O_3_ system, where Lu has the largest atomic number among the rare-earth elements (*RE*), the following three phases have been reported: Lu_3_Al_5_O_12_, LuAlO_3_, and Lu_4_Al_2_O_9_. These phases have been actively investigated as host materials, not only for phosphors (Ding *et al.*, 2011 ▸; Xiang *et al.*, 2016 ▸; Wang *et al.*, 2018 ▸), but also for scintillators, owing to their large radiation absorption cross sections arising from the presence of Lu. Various scintillation properties of Ce- and Pr-doped Lu_3_Al_5_O_12_ and LuAlO_3_ crystals have been characterized (Wojtowicz, 1999 ▸; Nikl, 2000 ▸; Wojtowicz *et al.*, 2006 ▸; Nikl *et al.*, 2013 ▸), and the luminescence properties of Ce- and Pr-doped Lu_4_Al_2_O_9_ evaluated (Lempicki *et al.*, 1996 ▸; Zhang *et al.*, 1997 ▸, Zhang *et al.*, 1998 ▸; Drozdowski *et al.*, 2005 ▸). The crystal structures of the lutetium aluminates Lu_3_Al_5_O_12_ (Euler & Bruce, 1965 ▸) and LuAlO_3_ (Dernier & Maines, 1971 ▸; Shishido *et al.*, 1995 ▸) have been determined as garnet-type (LUAG) and perovskite-type (LUAP), respectively. However, to date, there have been no reports of the lattice constants of Lu_4_Al_2_O_9_, although Shirvinskaya & Popova (1977 ▸) treated it as isotypic with Y_4_Al_2_O_9_ and have reported the *d*-spacings and relative peak intensities in the powder X-ray diffraction pattern (PDF#00-033-0844).

Many *RE*Al_2_O_9_ compounds have been investigated in detail. After Warshaw & Roy (1959 ▸) first reported the existence of Y_4_Al_2_O_9_, Reed & Chase (1962 ▸) determined the space group of this material as *P*2_1_/*c* using X-ray Weissenberg and precession photography. Christensen & Hazell (1991 ▸) later determined the crystal structure of Y_4_Al_2_O_9_ using powder synchrotron X-ray and neutron diffraction. Brandle & Steinfink (1969 ▸) also prepared crystals of *RE*Al_2_O_9_ (*RE* = Sm, Gd, Eu, Dy, Ho) and determined the crystal structure of Eu_4_Al_2_O_9_ using X-ray diffraction.

The lattice parameters of *RE*_4_Al_2_O_9_ have previously been reported for *RE* = Y (Lehmann *et al.*, 1987 ▸; Reed & Chase, 1962 ▸; Christensen & Hazell, 1991 ▸; Yamane *et al.*, 1995*b* ▸; Talik *et al.*, 2016 ▸), La (Dohrup *et al.*, 1996 ▸), Pr (Dohrup *et al.*, 1996 ▸), Nd (Dohrup *et al.*, 1996 ▸), Sm (Brandle & Steinfink, 1969 ▸; Mizuno *et al.*, 1977*a* ▸; Yamane *et al.*, 1995*a* ▸), Eu (Brandle & Steinfink, 1969 ▸; Mizuno *et al.*, 1977*b* ▸; Yamane *et al.*, 1995*a* ▸), Gd (Brandle & Steinfink, 1969 ▸; Mizuno *et al.*, 1977*b* ▸; Yamane *et al.*, 1995*a* ▸; Dohrup *et al.*, 1996 ▸; Martín-Sedeño *et al.*, 2006 ▸), Tb (Jero & Kriven, 1988 ▸; Yamane *et al.*, 1995*a* ▸; Dohrup *et al.*, 1996 ▸; Li *et al.*, 2009 ▸), Dy (Brandle & Steinfink, 1969 ▸; Mizuno *et al.*, 1978 ▸; Yamane *et al.*, 1995*a* ▸), Ho (Brandle & Steinfink, 1969 ▸; Mizuno, 1979 ▸; Yamane *et al.*, 1995*a* ▸), Er (Mizuno, 1979 ▸; Yamane *et al.*, 1995*a* ▸), Tm (Yamane *et al.*, 1995*a* ▸), and Yb (Mizuno & Noguchi, 1980 ▸; Yamane *et al.*, 1995*a* ▸).

Wu & Pelton (1992 ▸) investigated the phase diagram of the Lu_2_O_3_–Al_2_O_3_ system and showed that Lu_4_Al_2_O_9_ melted congruently at 2313 K under an inert atmosphere. Petrosyan *et al.* (2006 ▸) studied the same system under a reducing atmosphere and reported that Lu_4_Al_2_O_9_ could be formed by reaction of Lu_2_O_3_ and Lu_3_Al_5_O_12_ at 1923 K, but decomposed into Lu_2_O_3_ and a melt at 2273 K. Subsequently, Petrosyan *et al.* (2013 ▸) observed incongruent melting of Lu_4_Al_2_O_9_ at 2123 K under an Ar / 2% H_2_ atmosphere using differential thermal analysis (DTA). Klimm (2010 ▸) employed DTA to investigate LuAlO_3_ melting behavior in a 5 *N* pure Ar flow and concluded that the congruent and incongruent melting of LuAlO_3_ depended on the atmospheric conditions. The author also concluded that the phase diagram at around Lu:Al = 1:1 under an inert atmosphere, previously reported by Wu & Pelton (1992 ▸), is correct. Yamane *et al.* (1995*a* ▸) reported that only a very small amount of Lu_4_Al_2_O_9_ can be obtained by reactions in air at 1673–2073 K, even though *RE*_4_Al_2_O_9_ (*RE* = Y, Sm–Yb) can be synthesized under the same conditions.

Following these reports, the present authors also attempted to synthesize Lu_4_Al_2_O_9_ by heating a 2:1 molar ratio powder mixture of Lu_2_O_3_ and Al_2_O_3_ at 2073 K for 2 h in air, but the sample obtained was a mixture of LuAlO_3_ and Lu_2_O_3_ (see Fig. S1*a* in the supporting information). The method used to prepare the single crystals of Lu_4_Al_2_O_9_ used for the present diffraction study is described below.

## Structural commentary   

**Figure 1:**
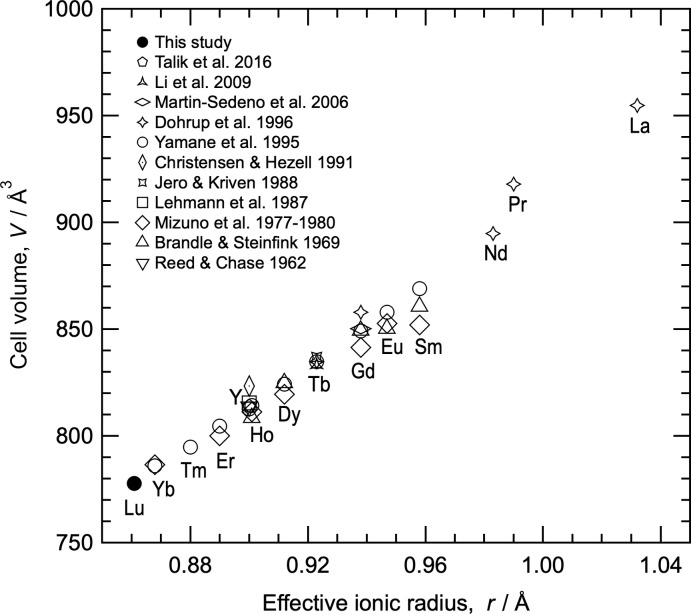
Unit-cell volume of *RE*_4_Al_2_O_9_ *versus* effective ionic radius for the trivalent rare-earth anion (*RE* ^3+^) with sixfold coordination.

X-ray diffraction spots from the Lu_4_Al_2_O_9_ single crystal were indexed on the basis of a monoclinic unit cell with lattice parameters: *a* = 7.236 (2) Å, *b* = 10.333 (2) Å, *c* = 11.096 (3) Å, and *β* = 108.38 (2)°. As shown in Fig. 1 ▸, the unit-cell volume of Lu_4_Al_2_O_9_ calculated from these parameters lies on the extrapolated line of *RE*_4_Al_2_O_9_ volumes plotted against the effective ionic radii for sixfold coordination of the trivalent rare-earth anions (*RE* ^3+^) (Shannon, 1976 ▸). In other words, Lu_4_Al_2_O_9_ containing Lu, which has the smallest effective ionic radius of the *RE* atoms, has the smallest unit-cell volume in the *RE*_4_Al_2_O_9_ series, in line with predictions arising from the lanthanide contraction.

**Figure 2:**
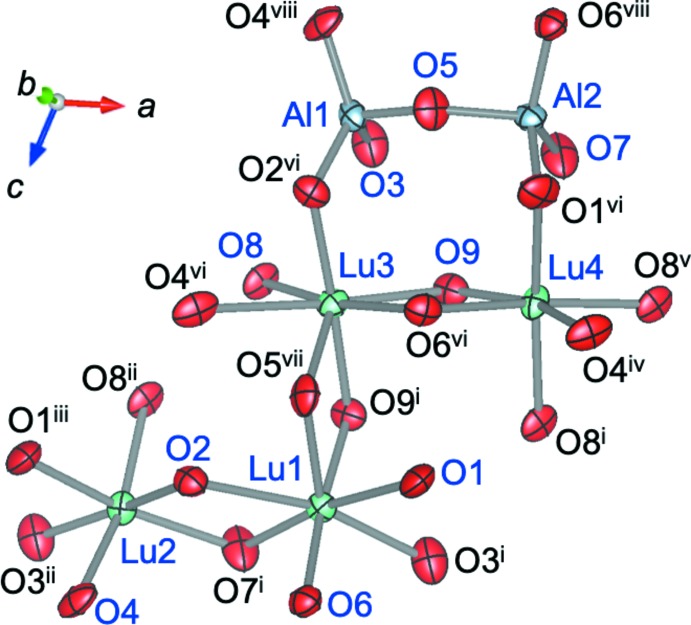
The atomic arrangement of Lu_4_Al_2_O_9_ depicted with displacement ellipsoids at the 99% probability level. [Symmetry codes: (i) 1 − *x*, −*y*, 1 − *z*; (ii) −*x*, −*y*, 1 − *z*; (iii) *x* − 1, *y*, *z*; (iv) 1 + *x*, −*y* + 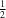, *z* − 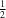; (v) *x* + 1, *y*, *z*; (vi) *x*, −*y* + 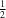, *z* − 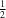; (vii) *x*, −*y* + 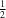, *z* + 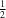; (viii) *x*, *y*, *z* − 1.]

**Figure 3:**
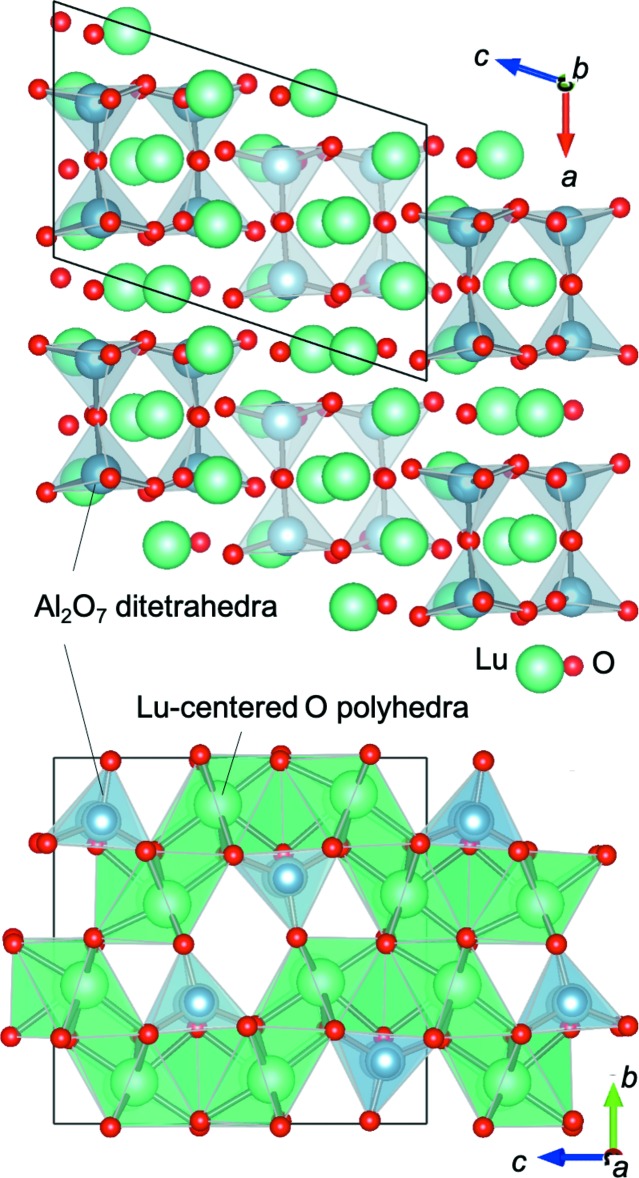
The crystal structure of Lu_4_Al_2_O_9_ highlighting the Al_2_O_7_ di­tetra­hedra viewed down the *b* axis (upper), and the Al_2_O_7_ di­tetra­hedra and Lu-centered polyhedra viewed down the *a* axis (lower).

The crystal structure of Lu_4_Al_2_O_9_ (space group *P*2_1_/*c*), determined using Eu_4_Al_2_O_9_ (Brandle & Steinfink, 1969 ▸) as the starting model, contains two crystallographically distinct Al sites, four Lu sites, and nine O sites. The two Al sites are tetra­hedrally coordinated by oxygen atoms. The two Al tetra­hedra are connected through a shared O5 atom, forming an Al_2_O_7_ di­tetra­hedral oxy-aluminate group (Fig. 2 ▸). The Al_2_O_7_ dimers lie parallel to the *a* axis, and are related by the *c* glide symmetry operation (Fig. 3 ▸). The average Al1—O and Al2—O distances in Lu_4_Al_2_O_9_ are 1.744 and 1.756 Å, respectively, which are comparable to values found in Eu_4_Al_2_O_9_ (1.741 and 1.755 Å, Brandle & Steinfink, 1969 ▸) and Y_4_Al_2_O_9_ (1.739 and 1.769 Å, Lehmann *et al.*, 1987 ▸). The bond-valence sums (BVS; Brown & Altermatt, 1985 ▸) calculated using the Al—O distances and bond-valence parameters recently reported by Gagne & Hawthorne (2015 ▸) (*r*_0_ =1.634 Å, *b* = 0.39) are 3.02 and 2.93 for Al1 and Al2, respectively. These BVS values are close to those expected for trivalent Al. The Al1—O5—Al2 angle of the Al_2_O_7_ dimer is 134.9 (3)°, which is smaller than the corresponding angles in Eu_4_Al_2_O_9_ (141.9°; Brandle & Steinfink, 1969 ▸) and Y_4_Al_2_O_9_ (137.6°; Lehmann *et al.*, 1987 ▸).

Of the four crystallographically distinct Lu atoms, Lu1 and Lu3 are coordinated by seven oxygen atoms with five Lu—O distances in the range 2.219 (5)–2.344 (5) Å and two in the range 2.461 (6)–2.573 (6) Å. The remaining Lu atoms, Lu2 and Lu4, are coordinated by six oxygen atoms in distorted octa­hedra with Lu—O distances in the range 2.172 (6)–2.337 (6) Å.

The averages Lu—O distances for the six-fold coordinated Lu atoms in Lu_4_Al_2_O_9_ are 2.250 and 2.260 Å for Lu2 and Lu4, respectively. These values are 0.02–0.10 Å shorter than those for the LuO_6_ octa­hedra found in Lu_3_Al_5_O_12_ (2.352 Å; Euler & Bruce, 1965 ▸) and LuAlO_3_ (2.330 Å; Shishido *et al.*, 1995 ▸).

The average values for the Eu—O and Y—O distances in Eu_4_Al_2_O_9_ and Y_4_Al_2_O_9_ lie in the ranges 2.328–2.439 Å (Brandle & Steinfink, 1969 ▸) and 2.286–2.387 Å (Lehmann *et al.*, 1987 ▸), respectively. The differences between the *RE*—O lengths in *RE*_4_Al_2_O_9_ when *RE* = Eu and Lu (0.07–0.09 Å), and when *RE* = Y and Lu (0.02–0.05 Å) correspond to the differences between ^VI^ *r*_Eu_ − ^VI^ *r*_Lu_ (0.086 Å) and ^VI^ *r*_Y_ − ^VI^ *r*_Lu_ (0.039 Å), where ^VI^ *r*_Eu_, ^VI^ *r*_Lu_, and ^VI^ *r*_Lu_ are the effective ionic radii in sixfold coordination of Lu^3+^ (0.861 Å), Eu^3+^ (0.947 Å), and Y^3+^ (0.900 Å), respectively (Shannon, 1976 ▸). The BVS for Lu1, Lu2, Lu3, and Lu4, calculated using the bond-valence parameters (*r*_0_ = 1.939 Å, *b* = 0.403) of Gagné & Hawthorne (2015 ▸), are 2.766, 2.796, 2.642, and 2.714, respectively, which are smaller than the expected valence value of +3 for the Lu atoms. The polyhedral volumes of Lu1O_7_ (18.18 Å^3^), Lu2O_6_ (14.29 Å^3^), Lu3O_7_ (18.56 Å^3^), and Lu4O_6_ (14.24 Å^3^) are 1.1–1.7 Å^3^ and 0.5–0.8 Å^3^ smaller than those for Eu_4_Al_2_O_9_ (Eu1O_7_:19.85 Å^3^, Eu2O_6_:15.38 Å^3^, Eu3O_7_:20.14 Å^3^, and Eu4O_6_:15.71 Å^3^) and for Y_4_Al_2_O_9_ (Y1O_7_:18.66 Å^3^, Y2O_6_:14.77 Å^3^, Y3O_7_:19.33 Å^3^, and Y4O_6_:14.98 Å^3^), respectively. These differences in polyhedral volumes correlate with the differences in ionic radii of the lanthanides.

## Synthesis and crystallization   

The starting powders Al_2_O_3_ (Sumitomo Chemicals, AKP20, 99.99%) and Lu_2_O_3_ (Nippon Yttrium, 99.999%) were mixed in a molar ratio of Lu:Al = 2:1, ground with ethanol in an agate mortar, and pressed into a pellet. The pellet was placed in a BN crucible with an inner diameter of 18 mm and a height of 20 mm. The BN crucible was covered with a BN lid, and heated in a chamber with a carbon heater (Shimadzu Mectem, Inc., VESTA). The pellet was heated slowly under vacuum (∼10 ^−2^ Pa) from room temperature to 1273 K. During the 5 min. hold at 1273 K, the chamber was filled with Ar (99.9995%) up to 0.15 MPa. The temperature was then raised to 2173 K at a heating rate of 300 Kh^−1^. After being held at 2173 K for 4 h, the sample was cooled to 1473 K at a rate of 20 Kh^−1^, and then to room temperature by shutting off the heater. A part of the obtained sample was pulverized in the agate mortar, and powder X-ray diffraction measurements (Bruker D2 Phaser, Cu *Kα* radiation) confirmed that the major crystalline phase present in the sample was Lu_4_Al_2_O_9_, together with small amounts of LuAlO_3_ and unreacted Lu_2_O_3_ (Fig. S1*a*). Colorless crystals of Lu_4_Al_2_O_9_ were selected for single-crystal X-ray diffraction studies.

## Refinement   

Crystal data, data collection and structure refinement details are summarized in Table 1 ▸. The Eu atoms in the rare-earth metal sites in the structural model of Eu_4_Al_2_O_9_ (Brandle & Steinfink, 1969 ▸) were replaced by Lu atoms to generate the initial model. Several iterations of refinement yielded an *R* value of 10.07% and a residual electron density of ∼10 e Å^−3^. A subsequent refinement, performed by implementing the (100) twin plane observed in a study of Y_4_Al_2_O_9_ (Yamane *et al.*, 1995*b* ▸), yielded an *R*(*F* ^2^ > 2*σ*(*F* ^2^)) value of 1.92% with an approximate volume ratio of 6:4 for the twin domains.

## Supplementary Material

Crystal structure: contains datablock(s) I. DOI: 10.1107/S2056989020005757/cq2036sup1.cif

Structure factors: contains datablock(s) I. DOI: 10.1107/S2056989020005757/cq2036Isup2.hkl

Figure S1. Powder XRD patterns of samples prepared (a) under air and (b) under Ar. DOI: 10.1107/S2056989020005757/cq2036sup3.tif

CCDC reference: 1999289

Additional supporting information:  crystallographic information; 3D view; checkCIF report

## Figures and Tables

**Table 1:** Experimental details

Crystal data	
Chemical formula	Lu_4_Al_2_O_9_
*M*_r_	897.84
Crystal system, space group	Monoclinic, *P*2_1_/*c*
Temperature (K)	301
*a*, *b*, *c* (Å)	7.2360 (11), 10.3330 (19), 11.096 (3)
β (°)	108.381 (11)
*V* (Å^3^)	787.3 (3)
*Z*	4
Radiation type	Mo *K*α
μ (mm^−1^)	49.97
Crystal size (mm)	0.12 × 0.05 × 0.04

Data collection	
Diffractometer	Bruker D8 QUEST
Absorption correction	Multi-scan (*SADABS*; Bruker, 2016 ▸)
*T*_min_, *T*_max_	0.451, 0.746
No. of measured, independent and observed [*I* > 2σ(*I*)] reflections	32672, 2795, 2719
*R*_int_	0.035
(sin θ/λ)_max_ (Å^−1^)	0.748

Refinement	
*R*[*F* ^2^ > 2σ(*F* ^2^)], *wR*(*F* ^2^), *S*	0.019, 0.043, 1.17
No. of reflections	2795
No. of parameters	138
Δρ_max_, Δρ_min_ (e Å^−3^)	1.49, −1.81

Computer programs: *APEX3* (Bruker, 2018 ▸), *SAINT* (Bruker, 2017 ▸), *SHELXL2014/7* (Sheldrick, 2015 ▸), *VESTA* (Momma & Izumi, 2011 ▸) and *publCIF* (Westrip, 2010 ▸).

## supplementary crystallographic information

### Crystal data

**Table:**

Lu_4_Al_2_O_9_	*F*(000) = 1528
*M**_r_* = 897.84	*D*_x_ = 7.575 Mg m^−^^3^
Monoclinic, *P*2_1_/*c*	Mo *K*α radiation, λ = 0.71073 Å
*a* = 7.2360 (11) Å	Cell parameters from 1294 reflections
*b* = 10.3330 (19) Å	θ = 2.8–38.5°
*c* = 11.096 (3) Å	µ = 49.97 mm^−^^1^
β = 108.381 (11)°	*T* = 301 K
*V* = 787.3 (3) Å^3^	Chip, colourless
*Z* = 4	0.12 × 0.05 × 0.04 mm

### Data collection

**Table:**

Bruker D8 QUEST diffractometer	2719 reflections with *I* > 2σ(*I*)
Detector resolution: 7.3910 pixels mm^-1^	*R*_int_ = 0.035
ω and σcans	θ_max_ = 32.1°, θ_min_ = 2.8°
Absorption correction: multi-scan (SADABS; Bruker, 2016)	*h* = −10→10
*T*_min_ = 0.451, *T*_max_ = 0.746	*k* = −15→15
32672 measured reflections	*l* = −16→16
2795 independent reflections	

### Refinement

**Table:**

Refinement on *F*^2^	0 restraints
Least-squares matrix: full	*w* = 1/[σ^2^(*F*_o_^2^) + 17.273*P*] where *P* = (*F*_o_^2^ + 2*F*_c_^2^)/3
*R*[*F*^2^ > 2σ(*F*^2^)] = 0.019	(Δ/σ)_max_ = 0.001
*wR*(*F*^2^) = 0.043	Δρ_max_ = 1.49 e Å^−^^3^
*S* = 1.17	Δρ_min_ = −1.81 e Å^−^^3^
2795 reflections	Extinction correction: *SHELXL2014/7* (Sheldrick 2015b), Fc^*^=kFc[1+0.001xFc^2^λ^3^/sin(2θ)]^-1/4^
138 parameters	Extinction coefficient: 0.00026 (2)

### Special details

**Table:**

Geometry. All esds (except the esd in the dihedral angle between two l.s. planes) are estimated using the full covariance matrix. The cell esds are taken into account individually in the estimation of esds in distances, angles and torsion angles; correlations between esds in cell parameters are only used when they are defined by crystal symmetry. An approximate (isotropic) treatment of cell esds is used for estimating esds involving l.s. planes.
Refinement. Refined as a two-component inversion twin

### Fractional atomic coordinates and isotropic or equivalent isotropic displacement parameters (Å^2^)

**Table:**

	*x*	*y*	*z*	*U*_iso_*/*U*_eq_	
Al1	0.2142 (4)	0.1742 (2)	0.1270 (2)	0.0058 (4)	
Al2	0.6551 (4)	0.1717 (2)	0.1108 (2)	0.0059 (4)	
Lu1	0.52225 (7)	0.11375 (3)	0.78409 (2)	0.00572 (6)	
Lu2	0.02236 (6)	0.10027 (3)	0.80405 (2)	0.00574 (6)	
Lu3	0.34172 (7)	0.12783 (3)	0.44005 (2)	0.00605 (6)	
Lu4	0.83940 (6)	0.12082 (3)	0.41774 (3)	0.00610 (6)	
O1	0.7934 (8)	0.2450 (6)	0.7469 (5)	0.0102 (11)	
O2	0.2314 (8)	0.2439 (5)	0.7699 (5)	0.0072 (11)	
O3	0.2106 (13)	0.0095 (5)	0.1516 (5)	0.0102 (10)	
O4	0.0720 (8)	0.2340 (6)	0.9813 (6)	0.0092 (11)	
O5	0.4326 (10)	0.2381 (4)	0.1156 (5)	0.0085 (8)	
O6	0.6371 (8)	0.2328 (5)	0.9599 (5)	0.0072 (11)	
O7	0.6926 (13)	0.0084 (5)	0.1529 (5)	0.0111 (10)	
O8	0.0764 (12)	−0.0082 (5)	0.3927 (5)	0.0072 (9)	
O9	0.5643 (13)	0.0063 (5)	0.3906 (5)	0.0069 (9)	

### Atomic displacement parameters (Å^2^)

**Table:**

	*U*^11^	*U*^22^	*U*^33^	*U*^12^	*U*^13^	*U*^23^
Al1	0.0071 (12)	0.0044 (8)	0.0067 (9)	0.0012 (8)	0.0032 (8)	0.0005 (7)
Al2	0.0074 (12)	0.0050 (8)	0.0055 (8)	0.0010 (8)	0.0021 (8)	0.0012 (7)
Lu1	0.00622 (14)	0.00463 (12)	0.00594 (10)	0.00019 (12)	0.00140 (14)	−0.00101 (8)
Lu2	0.00566 (13)	0.00433 (12)	0.00733 (10)	−0.00031 (13)	0.00218 (14)	−0.00058 (8)
Lu3	0.00647 (13)	0.00518 (11)	0.00625 (10)	0.00024 (13)	0.00165 (13)	0.00101 (9)
Lu4	0.00538 (14)	0.00435 (10)	0.00863 (10)	0.00048 (13)	0.00231 (15)	0.00137 (9)
O1	0.008 (3)	0.013 (3)	0.008 (2)	0.0039 (19)	0.0002 (18)	0.002 (2)
O2	0.009 (3)	0.006 (2)	0.007 (2)	0.0012 (18)	0.0030 (19)	0.0010 (17)
O3	0.011 (3)	0.006 (2)	0.015 (2)	0.000 (3)	0.007 (3)	0.0006 (17)
O4	0.009 (3)	0.007 (2)	0.009 (2)	0.0039 (18)	−0.0009 (18)	−0.0003 (19)
O5	0.008 (2)	0.0064 (18)	0.012 (2)	−0.003 (2)	0.004 (2)	−0.0003 (15)
O6	0.008 (3)	0.007 (2)	0.007 (2)	0.0017 (18)	0.0018 (18)	0.0009 (17)
O7	0.013 (3)	0.008 (2)	0.016 (2)	0.003 (3)	0.009 (3)	0.0059 (18)
O8	0.005 (3)	0.006 (2)	0.009 (2)	−0.003 (2)	0.000 (3)	−0.0002 (16)
O9	0.010 (3)	0.0042 (19)	0.007 (2)	0.001 (3)	0.003 (3)	0.0001 (16)

### Geometric parameters (Å, º)

**Table:**

Al1—O3	1.724 (6)	Lu3—O5^iv^	2.310 (5)
Al1—O4^i^	1.733 (6)	Lu3—O6^ii^	2.529 (5)
Al1—O5	1.754 (7)	Lu3—O4^ii^	2.573 (6)
Al1—O2^ii^	1.767 (6)	Lu3—Al2^iv^	3.211 (2)
Al1—Lu1^ii^	3.219 (2)	Lu3—Al1^iv^	3.247 (2)
Al1—Lu3^ii^	3.247 (2)	Lu3—Lu3^iii^	3.4748 (8)
Al1—Lu3	3.336 (2)	Lu3—Lu4^iii^	3.4803 (7)
Al2—O7	1.749 (6)	Lu4—O4^ix^	2.198 (6)
Al2—O1^ii^	1.753 (6)	Lu4—O9	2.253 (8)
Al2—O6^i^	1.755 (6)	Lu4—O6^ii^	2.255 (6)
Al2—O5	1.767 (7)	Lu4—O8^v^	2.257 (8)
Al2—Lu3^ii^	3.211 (2)	Lu4—O1^ii^	2.287 (6)
Al2—Lu1^ii^	3.272 (2)	Lu4—O8^iii^	2.311 (5)
Al2—Lu4	3.285 (2)	Lu4—Lu3^iii^	3.4804 (7)
Lu1—O9^iii^	2.219 (5)	Lu4—Lu4^x^	3.5061 (8)
Lu1—O6	2.233 (5)	Lu4—Lu2^ix^	3.5641 (7)
Lu1—O3^iii^	2.236 (8)	Lu4—Lu3^v^	3.5652 (7)
Lu1—O7^iii^	2.277 (8)	Lu4—Lu1^ii^	3.5836 (7)
Lu1—O5^iv^	2.344 (5)	O1—Al2^iv^	1.753 (6)
Lu1—O2	2.461 (6)	O1—Lu2^v^	2.172 (6)
Lu1—O1	2.524 (6)	O1—Lu4^iv^	2.287 (6)
Lu1—Al1^iv^	3.219 (2)	O2—Al1^iv^	1.767 (6)
Lu1—Al2^iv^	3.272 (2)	O2—Lu3^iv^	2.238 (5)
Lu1—Lu2^v^	3.5579 (7)	O3—Lu2^vii^	2.213 (8)
Lu1—Lu4^iv^	3.5836 (7)	O3—Lu1^iii^	2.236 (8)
Lu1—Lu3	3.6270 (9)	O4—Al1^xi^	1.733 (6)
Lu2—O1^vi^	2.172 (6)	O4—Lu4^viii^	2.198 (6)
Lu2—O3^vii^	2.213 (8)	O4—Lu3^iv^	2.573 (6)
Lu2—O2	2.235 (6)	O5—Lu3^ii^	2.310 (5)
Lu2—O7^iii^	2.263 (8)	O5—Lu1^ii^	2.344 (5)
Lu2—O8^vii^	2.280 (5)	O6—Al2^xi^	1.754 (6)
Lu2—O4	2.337 (6)	O6—Lu4^iv^	2.255 (6)
Lu2—Lu1^vi^	3.5579 (7)	O6—Lu3^iv^	2.529 (5)
Lu2—Lu4^viii^	3.5641 (7)	O7—Lu2^iii^	2.263 (8)
Lu2—Lu3^iv^	3.6485 (7)	O7—Lu1^iii^	2.277 (8)
Lu2—Lu4^iii^	3.7187 (7)	O8—Lu4^vi^	2.257 (8)
Lu2—Lu3^vii^	3.9156 (7)	O8—Lu2^vii^	2.280 (5)
Lu3—O2^ii^	2.238 (5)	O8—Lu4^iii^	2.311 (5)
Lu3—O9	2.242 (8)	O9—Lu1^iii^	2.219 (5)
Lu3—O9^iii^	2.260 (5)	O9—Lu3^iii^	2.260 (5)
Lu3—O8	2.302 (7)		
			
O3—Al1—O4^i^	117.7 (3)	O2^ii^—Lu3—O8	96.84 (19)
O3—Al1—O5	116.2 (4)	O9—Lu3—O8	102.35 (18)
O4^i^—Al1—O5	94.7 (3)	O9^iii^—Lu3—O8	80.0 (2)
O3—Al1—O2^ii^	109.3 (3)	O2^ii^—Lu3—O5^iv^	106.69 (18)
O4^i^—Al1—O2^ii^	121.3 (3)	O9—Lu3—O5^iv^	120.4 (2)
O5—Al1—O2^ii^	94.3 (3)	O9^iii^—Lu3—O5^iv^	74.68 (16)
O3—Al1—Lu1^ii^	128.9 (3)	O8—Lu3—O5^iv^	123.6 (2)
O4^i^—Al1—Lu1^ii^	111.6 (2)	O2^ii^—Lu3—O6^ii^	78.65 (19)
O5—Al1—Lu1^ii^	45.30 (17)	O9—Lu3—O6^ii^	71.8 (2)
O2^ii^—Al1—Lu1^ii^	49.24 (19)	O9^iii^—Lu3—O6^ii^	104.6 (2)
O3—Al1—Lu3^ii^	138.1 (3)	O8—Lu3—O6^ii^	171.4 (2)
O4^i^—Al1—Lu3^ii^	52.0 (2)	O5^iv^—Lu3—O6^ii^	65.0 (2)
O5—Al1—Lu3^ii^	43.34 (18)	O2^ii^—Lu3—O4^ii^	74.48 (19)
O2^ii^—Al1—Lu3^ii^	108.5 (2)	O9—Lu3—O4^ii^	176.2 (2)
Lu1^ii^—Al1—Lu3^ii^	68.24 (5)	O9^iii^—Lu3—O4^ii^	103.8 (2)
O3—Al1—Lu3	72.9 (2)	O8—Lu3—O4^ii^	75.8 (2)
O4^i^—Al1—Lu3	155.5 (2)	O5^iv^—Lu3—O4^ii^	63.1 (2)
O5—Al1—Lu3	99.75 (19)	O6^ii^—Lu3—O4^ii^	109.64 (15)
O2^ii^—Al1—Lu3	38.37 (18)	O2^ii^—Lu3—Al2^iv^	96.50 (15)
Lu1^ii^—Al1—Lu3	67.47 (5)	O9—Lu3—Al2^iv^	94.44 (18)
Lu3^ii^—Al1—Lu3	135.61 (8)	O9^iii^—Lu3—Al2^iv^	86.21 (17)
O7—Al2—O1^ii^	104.2 (3)	O8—Lu3—Al2^iv^	155.68 (16)
O7—Al2—O6^i^	124.1 (3)	O5^iv^—Lu3—Al2^iv^	32.39 (17)
O1^ii^—Al2—O6^i^	119.7 (3)	O6^ii^—Lu3—Al2^iv^	32.95 (13)
O7—Al2—O5	115.6 (4)	O4^ii^—Lu3—Al2^iv^	88.36 (14)
O1^ii^—Al2—O5	93.5 (3)	O2^ii^—Lu3—Al1^iv^	93.90 (15)
O6^i^—Al2—O5	95.4 (3)	O9—Lu3—Al1^iv^	151.60 (17)
O7—Al2—Lu3^ii^	143.2 (3)	O9^iii^—Lu3—Al1^iv^	85.82 (16)
O1^ii^—Al2—Lu3^ii^	107.2 (2)	O8—Lu3—Al1^iv^	98.38 (18)
O6^i^—Al2—Lu3^ii^	51.62 (19)	O5^iv^—Lu3—Al1^iv^	31.41 (17)
O5—Al2—Lu3^ii^	44.47 (16)	O6^ii^—Lu3—Al1^iv^	89.32 (13)
O7—Al2—Lu1^ii^	123.1 (2)	O4^ii^—Lu3—Al1^iv^	32.07 (14)
O1^ii^—Al2—Lu1^ii^	49.8 (2)	Al2^iv^—Lu3—Al1^iv^	60.46 (5)
O6^i^—Al2—Lu1^ii^	111.7 (2)	O2^ii^—Lu3—Al1	29.36 (15)
O5—Al2—Lu1^ii^	43.89 (17)	O9—Lu3—Al1	79.05 (14)
Lu3^ii^—Al2—Lu1^ii^	68.03 (5)	O9^iii^—Lu3—Al1	149.97 (14)
O7—Al2—Lu4	65.8 (2)	O8—Lu3—Al1	85.02 (13)
O1^ii^—Al2—Lu4	41.4 (2)	O5^iv^—Lu3—Al1	134.72 (12)
O6^i^—Al2—Lu4	158.5 (2)	O6^ii^—Lu3—Al1	87.58 (13)
O5—Al2—Lu4	96.12 (18)	O4^ii^—Lu3—Al1	97.42 (14)
Lu3^ii^—Al2—Lu4	134.03 (8)	Al2^iv^—Lu3—Al1	115.70 (7)
Lu1^ii^—Al2—Lu4	66.26 (5)	Al1^iv^—Lu3—Al1	122.25 (5)
O9^iii^—Lu1—O6	174.8 (3)	O2^ii^—Lu3—Lu3^iii^	142.19 (14)
O9^iii^—Lu1—O3^iii^	86.4 (2)	O9—Lu3—Lu3^iii^	39.67 (13)
O6—Lu1—O3^iii^	89.4 (2)	O9^iii^—Lu3—Lu3^iii^	39.30 (19)
O9^iii^—Lu1—O7^iii^	85.8 (2)	O8—Lu3—Lu3^iii^	91.43 (16)
O6—Lu1—O7^iii^	98.0 (2)	O5^iv^—Lu3—Lu3^iii^	98.91 (14)
O3^iii^—Lu1—O7^iii^	101.03 (18)	O6^ii^—Lu3—Lu3^iii^	87.86 (13)
O9^iii^—Lu1—O5^iv^	74.76 (17)	O4^ii^—Lu3—Lu3^iii^	143.06 (13)
O6—Lu1—O5^iv^	105.69 (19)	Al2^iv^—Lu3—Lu3^iii^	90.40 (5)
O3^iii^—Lu1—O5^iv^	128.4 (2)	Al1^iv^—Lu3—Lu3^iii^	121.35 (4)
O7^iii^—Lu1—O5^iv^	124.3 (2)	Al1—Lu3—Lu3^iii^	116.13 (4)
O9^iii^—Lu1—O2	104.5 (2)	O2^ii^—Lu3—Lu4^iii^	137.27 (15)
O6—Lu1—O2	80.21 (19)	O9—Lu3—Lu4^iii^	95.71 (15)
O3^iii^—Lu1—O2	165.4 (2)	O9^iii^—Lu3—Lu4^iii^	39.5 (2)
O7^iii^—Lu1—O2	70.7 (2)	O8—Lu3—Lu4^iii^	41.12 (12)
O5^iv^—Lu1—O2	64.9 (2)	O5^iv^—Lu3—Lu4^iii^	96.21 (14)
O9^iii^—Lu1—O1	100.2 (2)	O6^ii^—Lu3—Lu4^iii^	144.07 (12)
O6—Lu1—O1	75.68 (19)	O4^ii^—Lu3—Lu4^iii^	85.06 (13)
O3^iii^—Lu1—O1	73.7 (2)	Al2^iv^—Lu3—Lu4^iii^	120.37 (4)
O7^iii^—Lu1—O1	171.6 (2)	Al1^iv^—Lu3—Lu4^iii^	87.25 (5)
O5^iv^—Lu1—O1	63.4 (2)	Al1—Lu3—Lu4^iii^	123.92 (4)
O2—Lu1—O1	112.92 (16)	Lu3^iii^—Lu3—Lu4^iii^	64.036 (14)
O9^iii^—Lu1—Al1^iv^	87.18 (17)	O4^ix^—Lu4—O9	163.0 (2)
O6—Lu1—Al1^iv^	95.83 (15)	O4^ix^—Lu4—O6^ii^	87.53 (18)
O3^iii^—Lu1—Al1^iv^	160.44 (18)	O9—Lu4—O6^ii^	77.1 (2)
O7^iii^—Lu1—Al1^iv^	96.91 (18)	O4^ix^—Lu4—O8^v^	84.7 (2)
O5^iv^—Lu1—Al1^iv^	32.14 (17)	O9—Lu4—O8^v^	110.30 (17)
O2—Lu1—Al1^iv^	32.95 (13)	O6^ii^—Lu4—O8^v^	171.9 (2)
O1—Lu1—Al1^iv^	89.26 (14)	O4^ix^—Lu4—O1^ii^	75.4 (2)
O9^iii^—Lu1—Al2^iv^	85.38 (16)	O9—Lu4—O1^ii^	108.45 (19)
O6—Lu1—Al2^iv^	92.47 (15)	O6^ii^—Lu4—O1^ii^	80.3 (2)
O3^iii^—Lu1—Al2^iv^	100.91 (19)	O8^v^—Lu4—O1^ii^	100.0 (2)
O7^iii^—Lu1—Al2^iv^	155.76 (18)	O4^ix^—Lu4—O8^iii^	95.5 (2)
O5^iv^—Lu1—Al2^iv^	31.50 (17)	O9—Lu4—O8^iii^	79.9 (2)
O2—Lu1—Al2^iv^	89.77 (14)	O6^ii^—Lu4—O8^iii^	98.7 (2)
O1—Lu1—Al2^iv^	32.02 (14)	O8^v^—Lu4—O8^iii^	79.7 (3)
Al1^iv^—Lu1—Al2^iv^	60.13 (5)	O1^ii^—Lu4—O8^iii^	170.9 (2)
O9^iii^—Lu1—Lu2^v^	91.9 (2)	O4^ix^—Lu4—Al2	104.05 (15)
O6—Lu1—Lu2^v^	82.89 (14)	O9—Lu4—Al2	83.86 (13)
O3^iii^—Lu1—Lu2^v^	36.67 (18)	O6^ii^—Lu4—Al2	91.72 (14)
O7^iii^—Lu1—Lu2^v^	137.63 (18)	O8^v^—Lu4—Al2	92.45 (14)
O5^iv^—Lu1—Lu2^v^	95.47 (17)	O1^ii^—Lu4—Al2	30.48 (15)
O2—Lu1—Lu2^v^	149.10 (13)	O8^iii^—Lu4—Al2	158.20 (14)
O1—Lu1—Lu2^v^	37.17 (13)	O4^ix^—Lu4—Lu3^iii^	135.90 (15)
Al1^iv^—Lu1—Lu2^v^	125.28 (5)	O9—Lu4—Lu3^iii^	39.61 (13)
Al2^iv^—Lu1—Lu2^v^	65.26 (5)	O6^ii^—Lu4—Lu3^iii^	92.25 (14)
O9^iii^—Lu1—Lu4^iv^	139.2 (2)	O8^v^—Lu4—Lu3^iii^	91.67 (16)
O6—Lu1—Lu4^iv^	37.22 (14)	O1^ii^—Lu4—Lu3^iii^	147.82 (15)
O3^iii^—Lu1—Lu4^iv^	85.85 (17)	O8^iii^—Lu4—Lu3^iii^	40.93 (18)
O7^iii^—Lu1—Lu4^iv^	135.01 (15)	Al2—Lu4—Lu3^iii^	120.03 (4)
O5^iv^—Lu1—Lu4^iv^	79.07 (14)	O4^ix^—Lu4—Lu4^x^	90.25 (15)
O2—Lu1—Lu4^iv^	91.80 (13)	O9—Lu4—Lu4^x^	96.21 (15)
O1—Lu1—Lu4^iv^	39.37 (13)	O6^ii^—Lu4—Lu4^x^	137.47 (14)
Al1^iv^—Lu1—Lu4^iv^	86.95 (5)	O8^v^—Lu4—Lu4^x^	40.44 (13)
Al2^iv^—Lu1—Lu4^iv^	57.05 (4)	O1^ii^—Lu4—Lu4^x^	139.71 (15)
Lu2^v^—Lu1—Lu4^iv^	59.875 (14)	O8^iii^—Lu4—Lu4^x^	39.3 (2)
O9^iii^—Lu1—Lu3	36.30 (13)	Al2—Lu4—Lu4^x^	129.75 (4)
O6—Lu1—Lu3	143.97 (15)	Lu3^iii^—Lu4—Lu4^x^	61.365 (15)
O3^iii^—Lu1—Lu3	110.32 (15)	O4^ix^—Lu4—Lu2^ix^	39.59 (15)
O7^iii^—Lu1—Lu3	106.94 (16)	O9—Lu4—Lu2^ix^	142.06 (14)
O5^iv^—Lu1—Lu3	38.47 (12)	O6^ii^—Lu4—Lu2^ix^	82.47 (14)
O2—Lu1—Lu3	83.91 (13)	O8^v^—Lu4—Lu2^ix^	93.01 (16)
O1—Lu1—Lu3	81.20 (13)	O1^ii^—Lu4—Lu2^ix^	35.84 (15)
Al1^iv^—Lu1—Lu3	56.26 (4)	O8^iii^—Lu4—Lu2^ix^	135.12 (17)
Al2^iv^—Lu1—Lu3	55.19 (4)	Al2—Lu4—Lu2^ix^	65.06 (4)
Lu2^v^—Lu1—Lu3	95.095 (18)	Lu3^iii^—Lu4—Lu2^ix^	172.933 (13)
Lu4^iv^—Lu1—Lu3	112.083 (15)	Lu4^x^—Lu4—Lu2^ix^	120.06 (2)
O1^vi^—Lu2—O3^vii^	81.6 (2)	O4^ix^—Lu4—Lu3^v^	45.83 (16)
O1^vi^—Lu2—O2	89.32 (19)	O9—Lu4—Lu3^v^	149.15 (16)
O3^vii^—Lu2—O2	169.1 (2)	O6^ii^—Lu4—Lu3^v^	133.35 (14)
O1^vi^—Lu2—O7^iii^	164.5 (2)	O8^v^—Lu4—Lu3^v^	39.02 (17)
O3^vii^—Lu2—O7^iii^	113.97 (17)	O1^ii^—Lu4—Lu3^v^	85.63 (15)
O2—Lu2—O7^iii^	75.2 (2)	O8^iii^—Lu4—Lu3^v^	88.7 (2)
O1^vi^—Lu2—O8^vii^	91.6 (2)	Al2—Lu4—Lu3^v^	98.07 (5)
O3^vii^—Lu2—O8^vii^	88.2 (2)	Lu3^iii^—Lu4—Lu3^v^	120.326 (15)
O2—Lu2—O8^vii^	98.0 (2)	Lu4^x^—Lu4—Lu3^v^	58.961 (16)
O7^iii^—Lu2—O8^vii^	89.1 (3)	Lu2^ix^—Lu4—Lu3^v^	61.562 (13)
O1^vi^—Lu2—O4	74.9 (2)	O4^ix^—Lu4—Lu1^ii^	86.32 (15)
O3^vii^—Lu2—O4	92.4 (2)	O9—Lu4—Lu1^ii^	85.60 (15)
O2—Lu2—O4	79.5 (2)	O6^ii^—Lu4—Lu1^ii^	36.81 (14)
O7^iii^—Lu2—O4	103.2 (2)	O8^v^—Lu4—Lu1^ii^	144.42 (14)
O8^vii^—Lu2—O4	166.2 (2)	O1^ii^—Lu4—Lu1^ii^	44.44 (15)
O1^vi^—Lu2—Lu1^vi^	44.59 (16)	O8^iii^—Lu4—Lu1^ii^	135.46 (19)
O3^vii^—Lu2—Lu1^vi^	37.11 (17)	Al2—Lu4—Lu1^ii^	56.69 (4)
O2—Lu2—Lu1^vi^	133.86 (14)	Lu3^iii^—Lu4—Lu1^ii^	117.97 (2)
O7^iii^—Lu2—Lu1^vi^	150.92 (17)	Lu4^x^—Lu4—Lu1^ii^	173.404 (16)
O8^vii^—Lu2—Lu1^vi^	87.2 (2)	Lu2^ix^—Lu4—Lu1^ii^	59.705 (14)
O4—Lu2—Lu1^vi^	84.98 (14)	Lu3^v^—Lu4—Lu1^ii^	121.266 (15)
O1^vi^—Lu2—Lu4^viii^	38.06 (15)	Al2^iv^—O1—Lu2^v^	139.9 (3)
O3^vii^—Lu2—Lu4^viii^	86.66 (16)	Al2^iv^—O1—Lu4^iv^	108.1 (3)
O2—Lu2—Lu4^viii^	82.52 (14)	Lu2^v^—O1—Lu4^iv^	106.1 (2)
O7^iii^—Lu2—Lu4^viii^	137.89 (15)	Al2^iv^—O1—Lu1	98.2 (3)
O8^vii^—Lu2—Lu4^viii^	129.58 (17)	Lu2^v^—O1—Lu1	98.2 (2)
O4—Lu2—Lu4^viii^	36.84 (14)	Lu4^iv^—O1—Lu1	96.2 (2)
Lu1^vi^—Lu2—Lu4^viii^	60.420 (13)	Al1^iv^—O2—Lu2	127.6 (3)
O1^vi^—Lu2—Lu3^iv^	85.18 (17)	Al1^iv^—O2—Lu3^iv^	112.3 (3)
O3^vii^—Lu2—Lu3^iv^	136.93 (14)	Lu2—O2—Lu3^iv^	109.3 (2)
O2—Lu2—Lu3^iv^	35.36 (14)	Al1^iv^—O2—Lu1	97.8 (2)
O7^iii^—Lu2—Lu3^iv^	83.02 (17)	Lu2—O2—Lu1	103.7 (2)
O8^vii^—Lu2—Lu3^iv^	133.14 (18)	Lu3^iv^—O2—Lu1	101.5 (2)
O4—Lu2—Lu3^iv^	44.56 (15)	Al1—O3—Lu2^vii^	126.3 (5)
Lu1^vi^—Lu2—Lu3^iv^	119.653 (15)	Al1—O3—Lu1^iii^	123.9 (5)
Lu4^viii^—Lu2—Lu3^iv^	59.233 (14)	Lu2^vii^—O3—Lu1^iii^	106.2 (2)
O1^vi^—Lu2—Lu1	128.85 (16)	Al1^xi^—O4—Lu4^viii^	135.4 (3)
O3^vii^—Lu2—Lu1	149.48 (17)	Al1^xi^—O4—Lu2	117.6 (3)
O2—Lu2—Lu1	40.30 (14)	Lu4^viii^—O4—Lu2	103.6 (2)
O7^iii^—Lu2—Lu1	35.63 (17)	Al1^xi^—O4—Lu3^iv^	95.9 (3)
O8^vii^—Lu2—Lu1	88.6 (2)	Lu4^viii^—O4—Lu3^iv^	96.4 (2)
O4—Lu2—Lu1	97.74 (14)	Lu2—O4—Lu3^iv^	95.9 (2)
Lu1^vi^—Lu2—Lu1	172.025 (12)	Al1—O5—Al2	134.9 (3)
Lu4^viii^—Lu2—Lu1	118.072 (14)	Al1—O5—Lu3^ii^	105.3 (3)
Lu3^iv^—Lu2—Lu1	59.438 (13)	Al2—O5—Lu3^ii^	103.1 (3)
O1^vi^—Lu2—Lu4^iii^	124.70 (15)	Al1—O5—Lu1^ii^	102.6 (3)
O3^vii^—Lu2—Lu4^iii^	102.42 (15)	Al2—O5—Lu1^ii^	104.6 (3)
O2—Lu2—Lu4^iii^	87.60 (14)	Lu3^ii^—O5—Lu1^ii^	102.39 (18)
O7^iii^—Lu2—Lu4^iii^	54.38 (15)	Al2^xi^—O6—Lu1	122.0 (3)
O8^vii^—Lu2—Lu4^iii^	34.8 (2)	Al2^xi^—O6—Lu4^iv^	125.6 (3)
O4—Lu2—Lu4^iii^	156.67 (14)	Lu1—O6—Lu4^iv^	106.0 (2)
Lu1^vi^—Lu2—Lu4^iii^	117.607 (14)	Al2^xi^—O6—Lu3^iv^	95.4 (2)
Lu4^viii^—Lu2—Lu4^iii^	159.792 (10)	Lu1—O6—Lu3^iv^	99.6 (2)
Lu3^iv^—Lu2—Lu4^iii^	118.642 (17)	Lu4^iv^—O6—Lu3^iv^	100.7 (2)
Lu1—Lu2—Lu4^iii^	60.716 (12)	Al2—O7—Lu2^iii^	126.1 (5)
O1^vi^—Lu2—Lu3^vii^	85.85 (16)	Al2—O7—Lu1^iii^	123.6 (5)
O3^vii^—Lu2—Lu3^vii^	56.75 (14)	Lu2^iii^—O7—Lu1^iii^	109.0 (2)
O2—Lu2—Lu3^vii^	128.77 (14)	Lu4^vi^—O8—Lu2^vii^	110.1 (3)
O7^iii^—Lu2—Lu3^vii^	102.54 (16)	Lu4^vi^—O8—Lu3	102.9 (2)
O8^vii^—Lu2—Lu3^vii^	31.5 (2)	Lu2^vii^—O8—Lu3	117.4 (3)
O4—Lu2—Lu3^vii^	146.03 (14)	Lu4^vi^—O8—Lu4^iii^	100.3 (3)
Lu1^vi^—Lu2—Lu3^vii^	62.045 (13)	Lu2^vii^—O8—Lu4^iii^	125.0 (2)
Lu4^viii^—Lu2—Lu3^vii^	119.182 (17)	Lu3—O8—Lu4^iii^	97.9 (2)
Lu3^iv^—Lu2—Lu3^vii^	161.782 (11)	Lu1^iii^—O9—Lu3	120.0 (4)
Lu1—Lu2—Lu3^vii^	116.038 (15)	Lu1^iii^—O9—Lu4	113.8 (3)
Lu4^iii^—Lu2—Lu3^vii^	55.607 (13)	Lu3—O9—Lu4	110.2 (2)
O2^ii^—Lu3—O9	102.6 (2)	Lu1^iii^—O9—Lu3^iii^	108.2 (2)
O2^ii^—Lu3—O9^iii^	176.7 (3)	Lu3—O9—Lu3^iii^	101.0 (3)
O9—Lu3—O9^iii^	79.0 (3)	Lu4—O9—Lu3^iii^	100.9 (3)

Symmetry codes: (i) *x*, *y*, *z*−1; (ii) *x*, −*y*+1/2, *z*−1/2; (iii) −*x*+1, −*y*, −*z*+1; (iv) *x*, −*y*+1/2, *z*+1/2; (v) *x*+1, *y*, *z*; (vi) *x*−1, *y*, *z*; (vii) −*x*, −*y*, −*z*+1; (viii) *x*−1, −*y*+1/2, *z*+1/2; (ix) *x*+1, −*y*+1/2, *z*−1/2; (x) −*x*+2, −*y*, −*z*+1; (xi) *x*, *y*, *z*+1.
