# Supplementary figures and images for: Crystal structure of lutetium aluminate (LUAM), Lu4Al2O9
Source: Acta Crystallogr E Crystallogr Commun. 2020 Apr 30;76(Pt 5):752–5. doi: 10.1107/S2056989020005757 (PMC7199276; doi:10.1107/S2056989020005757)

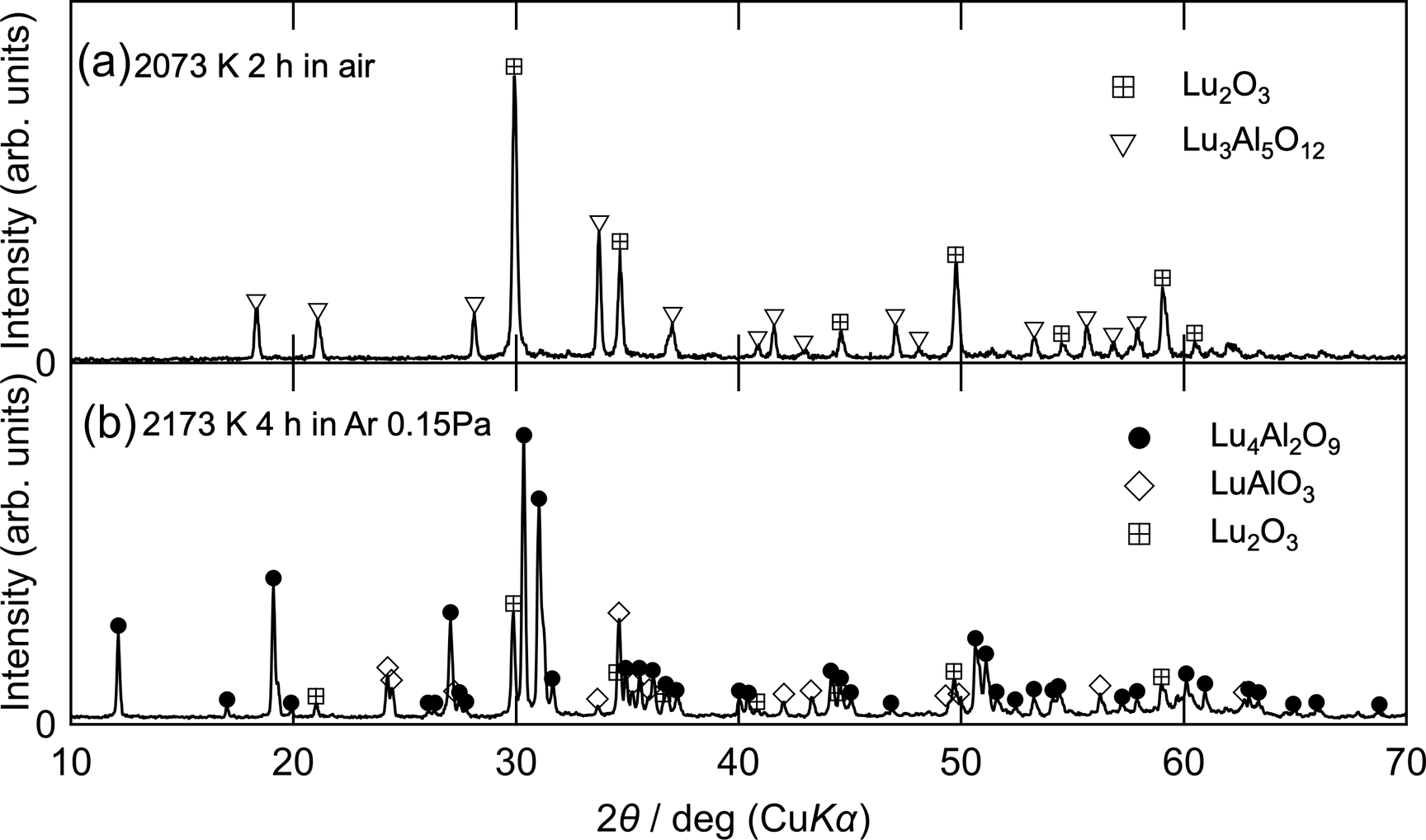

Supplement: Supplementary file 3 [file e-76-00752-sup3.tif]
